# Airborne Particulate Matter Size and Chronic Obstructive Pulmonary Disease Exacerbations: A Prospective, Risk-Factor Analysis Comparing Global Initiative for Obstructive Lung Disease 3 and 4 Categories

**DOI:** 10.3390/jpm13101505

**Published:** 2023-10-18

**Authors:** Gabriel-Petrică Bălă, Ovidiu Rosca, Felix Bratosin, Uday Shree Akkala Shetty, Sai Diksha Vutukuru, Isabella-Ionela Sanda, Monica Marc, Ovidiu Fira-Mladinescu, Cristian Oancea

**Affiliations:** 1 Center for Research and Innovation in Precision Medicine of Respiratory Diseases, “Victor Babes” University of Medicine and Pharmacy Timisoara, Eftimie Murgu Square 2, 300041 Timisoara, Romania; bala.gabriel@umft.ro (G.-P.B.); marc.monica@umft.ro (M.M.); mladinescu@umft.ro (O.F.-M.); 2 Doctoral School, “Victor Babes” University of Medicine and Pharmacy Timisoara, Eftimie Murgu Square 2, 300041 Timisoara, Romania; felix.bratosin@umft.ro (F.B.); isabella.sanda@umft.ro (I.-I.S.); oancea@umft.ro (C.O.); 3 Department of Infectious Diseases, “Victor Babes” University of Medicine and Pharmacy Timisoara, Eftimie Murgu Square 2, 300041 Timisoara, Romania; 4 Malla Reddy Institute of Medical Sciences, Suraram Main Road 138, Hyderabad 500055, India; udayshree98@gmail.com; 5 Department of General Medicine, MNR Medical College, Hyderabad 502285, India; dikshareddy611@gmail.com; 6 Discipline of Pulmonology, “Victor Babes” University of Medicine and Pharmacy Timisoara, Eftimie Murgu Square 2, 300041 Timisoara, Romania

**Keywords:** COPD, lung diseases, air pollutants, particulate matter

## Abstract

Current research primarily emphasizes the generalized correlations between airborne pollution and respiratory diseases, seldom considering the differential impacts of particular particulate matter sizes on chronic obstructive pulmonary disease (COPD) exacerbations in distinct Global Initiative for Obstructive Lung Disease (GOLD) categories. This study hypothesizes a critical association between particulate matter sizes (PM 1.0, PM 2.5, and PM 10) and exacerbation frequency in COPD patients categorized under GOLD 3 and GOLD 4, with a potential augmenting role played by proximity to main roads and industrial areas. This research aspires to offer a nuanced perspective on the exacerbation patterns in these groups, setting the stage for targeted intervention strategies. Utilizing a prospective design, this study followed 79 patients divided into GOLD 3 (*n* = 47) and GOLD 4 (*n* = 32) categories. The participants were monitored for ten days for daily activity levels, symptoms, living conditions, and airborne particulate matter concentrations, with spirometric evaluations employed to measure lung function. Statistical analyses were used to identify potential risk factors and significant associations. The analysis revealed substantial disparities in airborne particulate matter sizes between the two groups. The mean PM 1.0 concentration was notably higher in GOLD 4 patients (26 µg/m^3^) compared to GOLD 3 patients (18 µg/m^3^). Similarly, elevated PM 2.5 levels were observed in the GOLD 4 category (35 µg/m^3^) in contrast to the GOLD 3 category (24 µg/m^3^). A vital finding was the increased frequency of exacerbations in individuals residing within 200 m of main roads compared to those living further away (OR = 2.5, 95% CI: 1.5–4.1). Additionally, patients residing in homes smaller than 50 square meters demonstrated a greater frequency of exacerbations. Spirometry results corroborated the exacerbated condition in GOLD 4 patients, indicating a significant decline in lung function parameters compared to the GOLD 3 group. This study substantiates a significant association between airborne particulate matter sizes and exacerbation frequencies in COPD patients, particularly accentuating the increased risk in GOLD 4 patients. Our findings underscore the pivotal role of environmental factors, including the size of living areas and proximity to main roads, in influencing COPD exacerbations. These results suggest the need for personalized healthcare strategies and interventions, which account for environmental risk factors and the distinctions between GOLD 3 and GOLD 4 categories of COPD patients.

## 1. Introduction

Chronic obstructive pulmonary disease (COPD), a progressive lung condition characterized by persistent respiratory symptoms and airflow limitation, remains a significant public health concern globally [1,2]. The clinical course of COPD is often punctuated by exacerbations and acute episodes in which symptoms worsen significantly [3]. These exacerbations are primarily attributed to respiratory infections and environmental pollutants, prominently airborne particulate matter (PM) [4,5]. An increasing body of evidence underscores the varying impact of different particle sizes—PM 1.0, PM 2.5, and PM 10—on respiratory health [6]. These fine particles, with diameters of less than 10 μm, are capable of penetrating deep into the respiratory tract, inducing adverse respiratory responses, particularly in individuals with pre-existing pulmonary conditions [7].

The Global Initiative for Chronic Obstructive Lung Disease (GOLD) categorizes COPD patients into four groups: GOLD 1 to GOLD 4, based on the severity of their airflow limitation measured through spirometry [8]. GOLD 3 and GOLD 4, representing the severe and very severe stages of the disease, are characterized by a substantial decrease in forced expiratory volume in one second (FEV1) [9]. This stratification facilitates targeted interventions and management strategies, with a focus on preventing the frequent exacerbations that are common in these advanced stages of COPD.

Understanding the exacerbation patterns within the GOLD 3 and GOLD 4 categories requires comprehensive analytical tools. In this regard, the COPD Assessment Test (CAT) and the Modified British Medical Research Council (mMRC) dyspnea scale serve as pivotal tools [10,11]. The CAT, a patient-completed instrument, provides a measure of the health status of individuals with COPD, allowing for an assessment of the impact of the disease on the patient’s well-being [12,13,14]. On the other hand, the mMRC dyspnea scale facilitates the evaluation of the breathlessness experienced by COPD patients, a significant indicator of exacerbation severity and frequency [15].

Air pollution, particularly the fine and ultrafine particles categorized as PM 1.0, PM 2.5, and PM 10, poses a threat to COPD patients [16]. These particles are principally generated from combustion processes, including motor vehicle emissions, industrial activities, and residential heating [17]. Importantly, the size of these particles dictates their potential to infiltrate the lower respiratory tract. While larger particles (PM 10) generally become trapped in the upper airways, finer particles (PM 1.0 and PM 2.5) possess the capacity to penetrate deeper into the lungs, potentially causing more significant harm and exacerbating COPD symptoms [18,19].

The geographical location of individuals, especially their proximity to main roads and industrial areas, has been observed to correlate with the severity and frequency of COPD exacerbations [20,21,22]. Traffic-related air pollution, characterized by high levels of particulate matter, has been increasingly linked to respiratory morbidity and mortality [23,24,25]. Consequently, investigating the impact of these environmental factors, combined with particle size considerations, is vital in constructing a nuanced understanding of exacerbation patterns among COPD patients, particularly within the GOLD 3 and GOLD 4 categories.

Despite substantial research in this domain, a detailed analysis delineating the influence of specific particle sizes on COPD exacerbations within distinct GOLD categories remains sparse. The existing literature predominantly focuses on general correlations between air pollution and respiratory diseases, with limited studies segregating the effects based on the severity of COPD and the physical properties of PM [26,27]. This research seeks to fill this gap, providing a nuanced view of the interplay between airborne particulate matter size and exacerbation frequencies in GOLD 3 and GOLD 4 COPD patients.

Given this background, the present study hypothesizes that there exists a significant association between airborne particulate matter size (PM 1.0, PM 2.5, and PM 10) and the frequency of exacerbations among COPD patients categorized as GOLD 3 and GOLD 4. Furthermore, it is assumed that proximity to main roads and industrial areas serves as a substantial risk factor for increased exacerbation and severity of respiratory diseases [28]. This study aims to quantitatively analyze these relationships, offering insights into the exacerbation patterns within these specific patient groups, thereby paving the way for targeted intervention strategies. Through a prospective design, the study seeks to evaluate the risk factors contributing to exacerbation severity, focusing on the implications of particle size and COPD assessment scales, facilitating a deeper understanding and refined management strategies for COPD patients.

## 2. Materials and Methods

### 2.1. Study Design and Ethics

A systematic observational cohort study was undertaken to examine the potential associations between microparticulate air pollution, atmospheric determinants, and the frequency and severity of exacerbations in COPD patients. This investigation centered on individuals previously diagnosed with COPD, who were under the care of the Pulmonology Clinic at the “Victor Babes” Clinical Hospital located in Timisoara, Romania. This in-depth research was carried out over a span of seven months, specifically from September 2020 to March 2021, a timeframe that allowed for meticulous collection and analysis of relevant data points, while the measurement devices were installed in each patient’s home for a duration of ten days.

In accordance with the ethical guidelines delineated in the Declaration of Helsinki, the study underwent rigorous scrutiny and subsequently gained approval from the Institutional Review Board of the “Victor Babes” Clinical Hospital, documented under reference number 6111 on the date of August 18, 2020. Prior to initiating the study, a comprehensive process was undertaken to obtain informed consent from each participant. The study incorporated a strategy of home visits, where researchers engaged directly with participants in their respective residences. This approach facilitated a deeper understanding of the participants’ living conditions and their potential influence on COPD exacerbations and allowing for home measurements.

Furthermore, to accurately measure the impact of air pollution on COPD exacerbations, sophisticated air-pollution-monitoring equipment was installed at the residences of the participants. This facilitated real-time data acquisition of the various aspects of air quality, notably focusing on microparticulate concentrations. By integrating atmospheric factors into the analysis, the study aspired to uncover nuanced correlations between environmental variables and the exacerbation patterns seen in COPD patients classified as GOLD 3 and GOLD 4 in COPD severity.

### 2.2. Inclusion and Exclusion Criteria

The study selected a group of individuals diagnosed with severe or very severe COPD (stages 3 and 4 according to the GOLD classification, with an FEV1 < 50%) [29], primarily spending their time indoors and thereby consistently exposed to a stable air quality environment. The inclusion parameters were set as follows: participants aged 45 years or above, having a documented COPD diagnosis for at least a year, and exhibiting no acute exacerbations during the monitoring phase. Moreover, to align with the study’s focus on indoor air pollution, we also included individuals who reside within close proximity to urban areas to measure the distance from major roads or industrial areas, potentially experiencing heightened levels of particulate matter exposure.

The exclusion criteria were set to eliminate individuals who might introduce variability in the study outcome. This included individuals below 45 years of age, those presenting FEV1 values ≥ 50% or FEV1/FVC ratios ≥ 0.7, and those who had acute exacerbations during the monitoring period. Throughout the study, participants were observed to maintain stability in their health status, with adherence to medication regimes prescribed as per the GOLD guidelines, ensuring a homogenous and reliable data set for analysis.

### 2.3. Data Collection, Definitions, and Procedures

In this study, a structured approach was employed to extract data from patients’ electronic medical records and personal paper records. This process was undertaken meticulously by two researchers utilizing a standardized data collection form to ensure consistency and accuracy in the data compilation process.

To facilitate precise and reliable measurements of atmospheric parameters, the research utilized the state-of-the-art uRADMonitor SMOGGIE-PM, a product engineered by Magnasci SRL, headquartered in Timisoara, Romania. The attributes of this compact device, with dimensions measuring 42 × 43 × 27 mm^3^, enables comprehensive air quality monitoring, thereby being an integral tool in gauging indoor and outdoor pollution levels at the participants’ residences. Encased within a water-resistant plastic frame, the device boasts effortless installation capabilities, making it a feasible option for both indoor and outdoor settings.

Equipped with an array of high-precision laser sensors, the device is proficient in capturing detailed data on particulate matter concentrations, specifically PM 10, PM 2.5, and PM 1.0. In an effort to provide a holistic view of the atmospheric conditions, the device is further equipped with sensors capable of monitoring humidity, atmospheric pressure, and temperature, factors that could potentially influence air quality and respiratory health. Its power supply is ensured through a standard 5 V micro-USB power cord, facilitating easy setup and usage.

A noteworthy feature of the uRADMonitor SMOGGIE-PM is its connectivity capabilities, leveraging a WiFi connection to facilitate real-time data transmission [30]. These data can be accessed centrally via the uRADMonitor API, providing a centralized data repository, or alternatively, it can be viewed in a decentralized manner through local network connections. With a measurement frequency set at every 60 s, the device offers the advantage of real-time monitoring, calculating the minimum, maximum, and mean values of the recorded parameters during the observation period. This real-time data acquisition capability stands as a pivotal component in dissecting the intricate relationship between airborne particulate matter size and COPD exacerbations, especially when comparing the impacts across the GOLD 3 and GOLD 4 categories. According to the GOLD guidelines [10], COPD exacerbation is defined as an acute worsening of respiratory symptoms that results in additional therapy.

### 2.4. Study Variables

At the core of our study, a significant emphasis was placed on determining the respiratory symptoms and clinical conditions prevalent in COPD patients. Symptoms such as cough, dyspnea, wheezing, and chest constriction were meticulously documented, alongside a detailed record of comorbidities, including asthma, bronchiectasis, respiratory infections, hypertension, ischemic heart disease, heart failure, diabetes mellitus, cerebrovascular disease, hyperlipidemia, osteoporosis, and renal disease.

Concomitantly, the study also registered several biometric and physiological parameters, such as height, weight, and derived variables like body mass index (BMI) categorized into underweight (BMI < 18.5), normal weight (18.5 < BMI < 25), overweight (25 < BMI < 30), and obese (BMI > 30). Spirometry measurements encompassed parameters like forced vital capacity (FVC(L) and FVC(%)) and forced expiratory volume in the first second (FEV1(L) and FEV1(%)), alongside the GOLD categorization and the FEV1/FVC ratio. Additionally, forced expiratory flow at 25 to 75% (FEF 25/75(L) and FEF 25/75(%)) were noted, providing a detailed overview of the patients’ lung function.

Furthermore, patients were evaluated based on the COPD Assessment Test (CAT) [31], with scores recorded in individual categories (CAT1 to CAT8) and as a total score, supplemented with the Modified Medical Research Council (mMRC) dyspnea scale ratings [32]. A critical aspect of the study was the assessment of air quality and environmental conditions. Air quality parameters, such as PM 1.0, PM 2.5, and PM 10, along with temperature, atmospheric pressure, and humidity levels, were monitored meticulously, recording their maximum, minimum, and mean values over the period of study. These data were complemented by the installation and removal dates of monitoring devices, the number of monitoring days, and the specific device utilized, enhancing the granularity of the data collected.

Regarding demographic data, information on patients’ age, gender, and education levels spanning from elementary school to university degrees was collected. The study also encompassed an analysis of patients’ lifestyle and living conditions. This involved capturing data on smoking status, inclusive of pack years and second-hand smoke exposure, as well as physical activity levels categorized as less than 30 min, between 30 min to one hour, and more than one hour. The living conditions, including the home surface area and energy sources for cooking and heating, were documented. Moreover, residential features like urban or rural settings and proximity to the main road (categorized as <50 m, 50–200 m, and >200 m) were registered, establishing a potential link between residential locale and COPD exacerbations.

Lastly, medical laboratory parameters, such as fasting blood glucose, systolic and diastolic blood pressures, heart rate, and levels of creatinine and urea were recorded to provide a comprehensive view of the patients’ health status, constituting an integral component of the multifaceted analysis envisaged in this study aimed at assessing the dynamics of COPD exacerbations among GOLD 3 and GOLD 4 COPD patients.

### 2.5. Statistical Analysis

Data management and analysis were conducted utilizing the statistical software SPSS version 26.0 (SPSS Inc., Chicago, IL, USA). For analysis, patients were enrolled into two study groups based on their COPD severity (GOLD 3 and GOLD 4 categories). Continuous variables were represented as mean ± standard deviation (SD), while categorical variables were expressed in terms of frequencies and percentages. To analyze the changes in continuous variables, Student’s *t*-test was employed, and the Chi-squared test was utilized for the categorical variables. Multivariate analysis was performed to identify the risk factors associated with COPD exacerbations and was adjusted for confounding factors, such as age, smoking status, and the pack-year smoking amount. A *p*-value threshold of less than 0.05 was set for statistical significance. A Bonferroni correction was performed to account for multiple comparisons and adjust the significance threshold. All results were double-checked to ensure accuracy and reliability.

## 3. Results

A total of 79 patients were recruited and followed in the current study, stratified as the GOLD 3 category (*n* = 47), and 32 patients were classified as having GOLD 4 COPD severity. The average age was slightly higher in the GOLD 3 group (66.5 ± 9.1) compared to the GOLD 4 group (62.6 ± 9.4), although this difference was not statistically significant with a *p*-value of 0.068. The male population was notably higher in the GOLD 4 category at 84.4%, compared to 72.3% in the GOLD 3 category, though this difference was not significant either, with a *p*-value of 0.210. Education levels and smoking status were comparable between the two groups with no significant disparities noted, as evidenced by the *p*-values of 0.342 and 0.561, respectively.

An analysis of the daily activity level showed that a larger percentage of individuals in the GOLD 4 category engaged in less than 30 min of daily activity (53.1%) compared to the GOLD 3 group (36.2%), although this difference was not statistically significant with a *p*-value of 0.301. Similarly, signs and symptoms, such as cough, phlegm production, dyspnea, wheezing, and chest constriction were prevalently noticed in both groups with no statistically significant differences.

However, the analysis did identify significant differences in BMI distributions and certain comorbidities between the two groups. BMI was significantly lower in the GOLD 4 group, with 50% having a BMI between 18.5 and 25, compared to 27.7% in the GOLD 3 group, denoted by a *p*-value of 0.018. Moreover, the GOLD 4 group exhibited a significant higher incidence of pulmonary comorbidities (excluding COPD) at 59.4%, compared to 27.7% in the GOLD 3 group, a finding that was statistically significant with a *p*-value of 0.004. Cardiovascular comorbidities were more prevalent in the GOLD 3 group (93.6%) compared to the GOLD 4 group (78.1%), a difference which was statistically significant with a *p*-value of 0.042, as seen in Table 1. However, after the Bonferroni correction, it was determined that all *p*-values higher than 0.0029 remained statistically insignificant, such as the BMI difference and the proportions of cardiovascular and pulmonary comorbidities.

Table 2 describes the patients’ living conditions stratified by COPD severity. Regarding the place of residence, it was noted that a considerable proportion of GOLD 3 patients resided in urban areas (83.0%), as opposed to 68.8% in the GOLD 4 group. This difference, however, was not statistically significant as evidenced by a *p*-value of 0.138. An analysis of living area size illustrated that the mean living area was substantially larger for the GOLD 3 group (79.1 ± 42.4 m^2^) compared to the GOLD 4 group (61.3 ± 30.4 m^2^), a difference that was statistically significant (*p*-value = 0.044), which was rendered insignificant after Bonferroni correction. Moreover, a categorial comparison of living areas revealed a significant variation between the two groups, indicated by a *p*-value of 0.017, but without statistical significance with the Bonferroni analysis. Notably, a majority of the GOLD 3 group resided in spaces larger than 60 m^2^ (57.4%), in contrast to only 28.1% in the GOLD 4 group.

The aspect of proximity to the main road was also explored, revealing that the GOLD 3 group had a mean distance of 3823.4 ± 1517.2 m, markedly higher than the GOLD 4 group who resided at an average distance of 800.2 ± 209.5 m from the main road. This variation was highly significant with a *p*-value of less than 0.001. The categorial comparison, however, did not indicate a significant difference, with a *p*-value of 0.215. Building heights were comparably similar for both groups, with no significant variation noted (*p* = 0.598).

Furthermore, the study evaluated different sources used for cooking and heating in the residences of both groups. A large fraction of both groups used gas as a source of cooking, though a slightly higher percentage was observed in the GOLD 3 group (95.7%) compared to the GOLD 4 group (85.7%). The differences in cooking source types, however, were not statistically significant, as indicated by their respective *p*-values. In terms of heating sources, a significant difference was observed with the utilization of gas in that 83.0% of the GOLD 3 group relied on gas compared to only 42.6% in the GOLD 4 group, a discrepancy that was statistically significant with a *p*-value of 0.009. The usage of other heating sources, like electric and biomass, did not showcase significant differences between the two groups.

The data presented in Table 3 offer insight into the variations in airborne particulate matter of sizes PM 1.0, PM 2.5, and PM 10 µg/m^3^. A significant differentiation between the two groups was observed in the maximum and mean values of PM 1.0 concentrations, in which the GOLD 4 group exhibited higher values compared to the GOLD 3 group, with respective *p*-values of less than 0.001 and 0.006, suggesting a statistically significant difference in the concentration of PM 1.0 particles in the living environments of the two groups. However, the mean values were not significantly different, account by the Bonferroni correction of the *p*-value.

**Figure 1:**
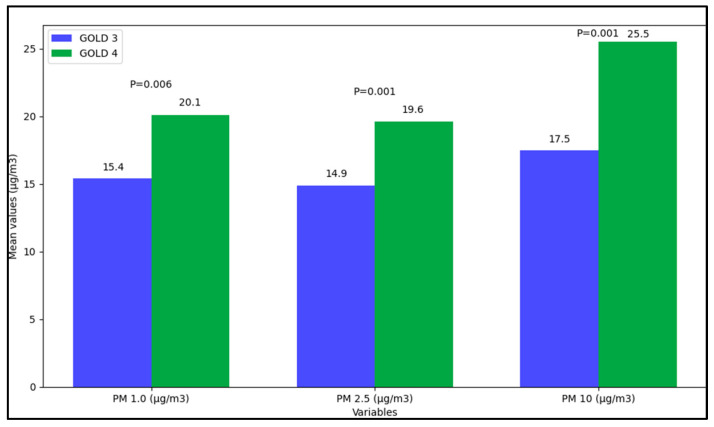
Home air measurements stratified by COPD severity.

Similarly, for PM 2.5 concentrations, the mean values were notably different, with the GOLD 4 group having a higher mean concentration (19.6 µg/m^3^) than the GOLD 3 group (14.9 µg/m^3^), which was statistically significant with a *p*-value of 0.001, as seen in Figure 1. The variations in minimum and maximum values, however, were not statistically significant, bearing *p*-values of 0.168 and 0.493, respectively. A pronounced difference was also noted in the PM 10 measurements, in which the GOLD 4 group demonstrated significantly higher maximum and mean values compared to the GOLD 3 group, with *p*-values of less than 0.001 in both cases. The minimum values exhibited no significant variation with a *p*-value of 0.227.

Regarding the environmental parameters within the homes, temperature readings across minimum, maximum, and mean values presented no substantial differences between the two groups, with *p*-values of 0.116, 0.304, and 0.660, respectively, indicating that temperature did not vary significantly between the two categories. Similarly, humidity levels were relatively consistent between both groups across all measures (minimum, maximum, and mean values), with *p*-values demonstrating no statistical significance (0.538, 0.092, and 0.274, respectively). Atmospheric pressure measurements within the homes also did not display a significant variation between the two groups in any of the noted parameters, with *p*-values of 0.319, 0.563, and 0.183 for minimum, maximum, and mean values, respectively.

The analysis of frequent exacerbations indicated a higher percentage of individuals in the GOLD 4 category (57.1%) experiencing recurrent exacerbations compared to the GOLD 3 category (38.3%), although this difference was not statistically significant with a *p*-value of 0.090. Similarly, the utilization of oxygen supplementation at home was somewhat more prevalent in the GOLD 4 category (62.9%) compared to GOLD 3 (57.4%), yet this disparity did not reach statistical significance as indicated by a *p*-value of 0.621, as presented in Table 4.

Significant differences were observed in the spirometry parameters between the two groups. Patients categorized under GOLD 4 demonstrated a marked decrease in various spirometry measures, such as FEV1 (both percentage and volume in liters), FVC (both percentage and volume in liters), the ratio of FEV1/FVC, and forced expiratory flow at 25–75% of the pulmonary volume (both in liters and percentage). All of these differences were highly significant with *p*-values of less than 0.001, illustrating a pronounced decline in lung function among patients in the GOLD 4 category when compared to those in the GOLD 3 category.

Regarding COPD assessment scores, the CAT scores were slightly higher in the GOLD 4 group (25.5 ± 7.4) compared to the GOLD 3 group (22.9 ± 6.5), suggesting a potentially worse quality of life, although the difference was not statistically significant, with a *p*-value of 0.103. The mMRC scores were also marginally higher in the GOLD 4 group, indicating a higher level of disability, but again, the difference was not statistically significant with a *p*-value of 0.065.

In evaluating the other studies, the analysis revealed that there were no significant differences in systolic and diastolic blood pressure, creatinine levels, and glucose levels between the two groups, with *p*-values of 0.512, 0.582, 0.079, and 0.297, respectively. Nevertheless, there was a notable difference in the blood urea nitrogen (BUN) levels between the groups, with the GOLD 3 group presenting higher levels compared to the GOLD 4 group, a difference that was statistically significant with a *p*-value of 0.024 but insignificant based according to the Bonferroni correction of the *p*-value at *p* < 0.0031.

**Figure 2:**
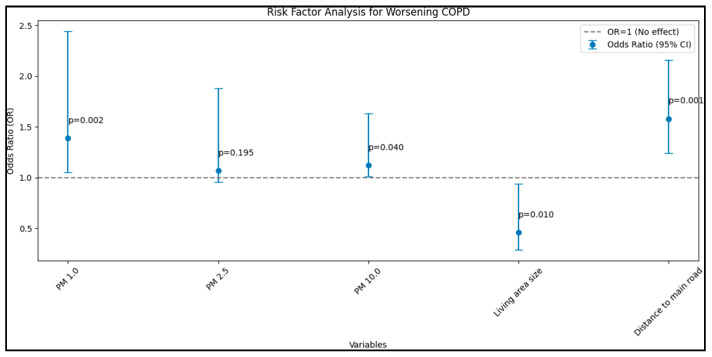
Risk-factor analysis for worsening COPD—GOLD as dependent variable.

Initially focusing on the GOLD as the dependent variable, the findings depicted a significant association between increasing PM 1.0 (µg/m^3^) concentrations and COPD exacerbation, illustrated by a β coefficient of 0.025 and an odds ratio (OR) of 1.39 (95% CI: 1.05–2.44, *p* = 0.002). Furthermore, a notable relationship was also evident with PM 10.0 (µg/m^3^) concentrations having a β coefficient of 0.016 and an OR of 1.12 (95% CI: 1.01–1.63, *p* = 0.040). Moreover, a vital correlation was discerned in relation to the distance to the main road, with a β coefficient of 0.033 and an OR of 1.58 (95% CI: 1.24–2.16, *p* < 0.001), indicating a heightened risk of COPD exacerbation for individuals residing closer to main roads. Conversely, living area size emerged as a protective factor, with an OR of 0.46 (95% CI: 0.29–0.94, *p* = 0.010), suggesting that larger living areas potentially mitigate the risk of COPD exacerbation, as seen in Figure 2.

**Figure 3:**
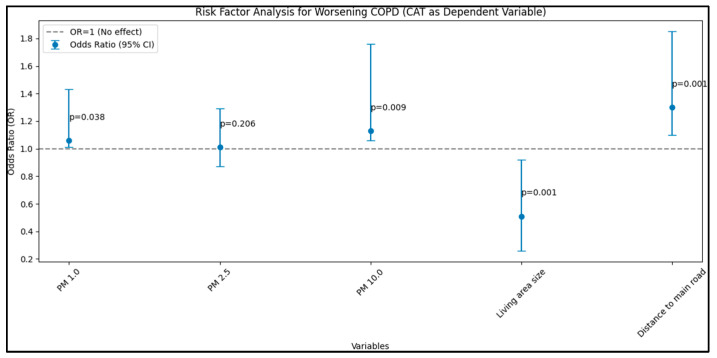
Risk-factor analysis for worsening COPD—CAT as dependent variable.

In considering the CAT as a dependent variable, a similar trend was observed in which PM 1.0 and PM 10.0 (µg/m^3^) displayed significant associations with higher CAT scores, indicative of worsened COPD symptoms. Noteworthy was the strong negative association between living area size and CAT scores, with an OR of 0.51 (95% CI: 0.26–0.92, *p* < 0.001), corroborating the protective effect noted in the GOLD-dependent analysis. Similarly, proximity to the main road was positively correlated with higher CAT scores, implying increased COPD symptoms (OR: 1.30, 95% CI: 1.10–1.85, *p* < 0.001), as presented in Figure 3.

**Figure 4:**
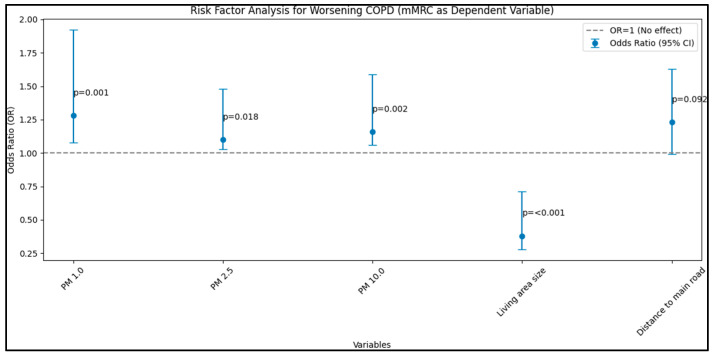
Risk-factor analysis for worsening COPD—mMRC as dependent variable.

Lastly, the analysis with mMRC as the dependent variable further substantiated the observed trends, with significant positive associations found between PM 1.0, PM 2.5, and PM 10.0 (µg/m^3^) concentrations and heightened mMRC scores, denoting exacerbated COPD symptoms. Once again, a larger living area size was inversely associated with mMRC scores, presenting a significant protective effect (OR: 0.38, 95% CI: 0.28–0.71, *p* < 0.001), as presented in Table 5 and Figure 4.

## 4. Discussion

### 4.1. Literature Findings

In the current investigation, a total of 79 patients with varied severity levels of COPD were meticulously examined to uncover potential disparities in demographic factors, daily activities, living conditions, and environmental elements between the GOLD 3 and GOLD 4 categories. Despite the non-significant variations in demographics and daily activities, the study identified pronounced disparities in the presence of certain comorbidities and BMI distributions between the groups. The GOLD 4 group exhibited lower BMI values and a higher incidence of pulmonary comorbidities (excluding COPD) compared to the GOLD 3 group, findings that were statistically significant. This may suggest a greater vulnerability to adverse health outcomes within the GOLD 4 cohort. The divergences in cardiovascular comorbidities, with a higher prevalence noted in the GOLD 3 group, warrants further exploration to understand the underlying causes and potential implications.

A comprehensive examination of living conditions illuminated significant differences between the two groups. The GOLD 3 group predominantly resided in urban areas and had considerably larger living spaces compared to the GOLD 4 group, a factor that emerged as statistically significant. Furthermore, a notable variation in proximity to main roads was uncovered, with the GOLD 3 group residing at a much greater distance compared to the GOLD 4 group, a difference that was highly significant. These findings may insinuate a potential link between living conditions and the severity of COPD, suggesting that individuals in the GOLD 4 category might be exposed to more environmental stressors due to their closer proximity to main roads.

Moreover, differences in airborne particulate matter within the living environments of the two groups were also prominent. The GOLD 4 category demonstrated significantly higher maximum and mean values for PM 1.0 and PM 2.5 concentrations compared to the GOLD 3 group, potentially indicating a higher level of environmental pollution exposure. Noteworthy was the stark contrast in PM 10 concentrations as well, further cementing the notion of heightened exposure to environmental contaminants within the GOLD 4 group. These variations propose a potential avenue of inquiry into the role of airborne particulates in exacerbating COPD symptoms and how they may contribute to the progression of the disease from GOLD 3 to GOLD 4.

Furthermore, the study scrutinized environmental parameters within the homes of both groups, revealing no significant differences in temperature, humidity, and atmospheric pressure measurements. This indicates that the indoor climatic conditions were relatively consistent across both groups. However, an analysis of exacerbations and the use of oxygen supplementation at home exhibited a higher tendency in the GOLD 4 group, although this was not statistically significant. Moreover, significant differences in spirometry parameters were detected between the two groups, with the GOLD 4 category illustrating a pronounced deterioration in lung function, pointing towards a substantial decline in respiratory health in this cohort.

When considering GOLD, CAT, and mMRC as dependent variables, the analysis revealed substantial associations between environmental factors and COPD exacerbations and symptoms. Particularly, increasing concentrations of PM 1.0 and PM 10.0 and proximity to main roads were positively associated with heightened COPD symptoms and exacerbations, while larger living area size emerged as a protective factor.

Recent research in respiratory medicine is steadily highlighting the link between chronic obstructive pulmonary disease (COPD) prevalence and increased exposure to particulate matter, especially PM 2.5 concentrations [33]. A growing body of the scientific literature is gradually uncovering the complex relationship between airborne particles and lung function. Notably, a recent study illustrated that a 7 µg/m^3^ increase in the 5-year mean particulate matter 10 (PM 10) interquartile range (IQR) correlated with a higher odds ratio (OR) of 1.33 in relation to COPD occurrence [34]. Interestingly, the data showed a close relationship in the odds ratios for PM 2.5 levels between >35 but <75 µg/m^3^ and PM 10 levels between >50 but <150 µg/m^3^, indicating a close connection in the negative respiratory effects of these particles. Moreover, it was observed that the odds did not significantly increase for PM 2.5 concentrations exceeding 75 µg/m^3^ compared to levels between 35 and 75 µg/m^3^, a finding that requires more detailed investigation.

In a prominent study conducted in Southern China, a significant association was found between exposure to ambient particulate matter, specifically PM 10/2.5, and the prevalence and progression of COPD [35]. The cross-sectional study analyzed data from almost 6000 patients, spanning over seven clusters selected randomly from four cities in Guangdong province. The study revealed considerable variations in COPD prevalence and atmospheric PM concentrations across the clusters, and a discernible correlation was established between heightened PM concentration levels and COPD prevalence, with an adjusted OR of 2.41 for PM 2.5 concentrations between >35 and ≤75 µg/m^3^ and 2.53 for concentrations exceeding 75 µg/m^3^, in comparison to levels of ≤35 µg/m^3^. Similarly, an adjusted OR of 2.44 was observed for PM1 concentrations between >50 and ≤150 µg/m^3^ compared to levels of ≤50 µg/m^3^. Moreover, an increase of 10 µg/m^3^ in PM 2.5 was associated with a 26 mL decrease in FEV1, a 28 mL average reduction in FVC, and a statistically significant 0.09% decline in the FEV1/FVC ratio. While similar associations were found with PM 10 concentrations, the correlations were slightly weaker. Consequently, other studies also concluded that higher levels of PM exposure were notably associated with an increase in COPD prevalence and a decline in respiratory function, emphasizing the critical need to further explore the repercussions of ambient particulate matter on respiratory health [36].

Similarly, several studies have revealed the harmful lung effects of prolonged exposure to vehicular emissions. One significant study showed that an increase of 2 µg/m^3^ in average PM 2.5 levels was linked to a decline of 13.5 mL in FEV1 and a continuous annual decrease by 2.1 mL [37]. This pattern was also noted in FVC declines, especially following long-term exposure to traffic-related emissions. In children, this increase was associated with decreased FVC values and a higher likelihood of FEV1 values falling below the expected 80% [38].

Furthermore, another study found that a 10 µg/m^3^ increase in average PM 2.5 levels was associated with a notable decrease in FEV1 percentages predicted, ranging from 0.09 to 1.5 units [39]. In a study focused on elderly men, the Normative Aging Study found that long-term exposure to black carbon—a major component of PM mainly from diesel emissions that was clearly linked with a faster decline in both FEV1 and FVC values [40]. Initially, the study found that the black carbon exposure had a stronger effect on FEV1, indicating a significant obstructive effect on the lungs. However, over time, a more restrictive pattern was seen, with FVC showing a greater decline compared to FEV1.

Adding to this, the ESCAPE meta-analysis investigated the possible obstructive effects associated with long-term PM 10 exposure. While the associations with COPD, identified by an FEV1/FVC ratio below 0.7, were not statistically significant, it pointed to the need for further, more extensive studies to better understand the complex dynamics and potential effects of long-term PM 10 exposure on lung health [41]. This ongoing discussion emphasizes the need for more comprehensive research to fully grasp the extent of ambient particulate matter’s impact on COPD pathophysiology.

### 4.2. Study Limitations

The study exhibited several limitations that should be noted when interpreting the results. Firstly, the investigation was geographically confined to the Western region of Romania, which might not fully represent the broader population with diverse environmental exposures. Secondly, the study exclusively focused on individuals classified with GOLD 3 and GOLD 4 severity stages of COPD, which might limit the generalizability of the findings to individuals with milder forms of the disease. Furthermore, the seven-month duration of the study might not be sufficiently extensive to fully encapsulate the possible seasonal variations in air pollution and its potential impacts on COPD exacerbations. The study’s reliance on technology for data acquisition, particularly the use of uRADMonitor SMOGGIE-PM, while advanced, may have technological limitations and potential inaccuracies that need to be considered. Moreover, the study took place when the COVID-19 pandemic peaked, from September 2020 to March 2021, which might affect the results somehow since traffic and pollution were much lower during the time of lockdowns [42]. Lastly, the study focused on patients who primarily spent their time indoors, which might not provide a complete picture of the effects of broader environmental variables on COPD exacerbations, including those experienced in outdoor settings or those engaged in different levels of physical activity.

## 5. Conclusions

This study offers initial insights into potential factors influencing COPD severity in patients categorized under GOLD 3 and GOLD 4. Notably, GOLD 4 individuals appear to be more exposed to higher PM concentrations, suggesting a potential link with worsened symptoms. Living closer to heavy traffic areas might aggravate symptoms, while larger living spaces could be protective. Given our limited sample size, these observations should be approached with caution and seen as preliminary, warranting further studies with larger cohorts.

## Figures and Tables

**Table 1:** Background characteristics stratified by COPD severity.

Variables *	GOLD 3 (*n* = 47)	GOLD 4 (*n* = 32)	*p*-Value
Age (mean ± SD)	66.5 ± 9.1	62.6 ± 9.4	0.068
Sex (male)	34 (72.3%)	27 (84.4%)	0.210
**Education**			0.342
Elementary school	12 (25.5%)	13 (40.6%)	
Junior and high school	28 (59.6%)	16 (50.0%)	
University degree	7 (14.9%)	3 (9.4%)	
**Smoking status**			0.561
Active smoker	13 (27.7%)	12 (37.5%)	
Former smoker	32 (68.1%)	18 (56.3%)	
Never smoker	2 (4.3%)	*n* (6.3%)	
Secondhand smoker	22 (46.8%)	15 (46.9%)	0.995
Pack years (mean ± SD)	39.7 ± 17.3	37.9 ± 17.1	0.649
**Level of daily activity**			0.301
<30 min	17 (36.2%)	17 (53.1%)	
30–60 min	16 (34.0%)	9 (28.1%)	
>60 min	14 (29.8%)	6 (18.8%)	
**Signs and symptoms**			
Cough	38 (80.9%)	23 (71.9%)	0.350
Phlegm	30 (63.8%)	19 (59.4%)	0.689
Dyspnea	32 (68.1%)	27 (84.4%)	0.102
Wheezing	20 (42.6%)	10 (31.3%)	0.309
Chest constriction	14 (29.8%)	10 (31.3%)	0.889
Number of COPD exacerbations (mean ± SD)	5.2 ± 2.1	7.4 ± 2.3	<0.001
**BMI (kg/m^2^)**			0.018
<18.5	2 (4.3%)	4 (12.5%)	
18.5–25	13 (27.7%)	16 (50.0%)	
25–30	13 (27.7%)	7 (21.9%)	
>30	19 (40.4%)	5 (15.6%)	
**Comorbidities**			
Cardiovascular	44 (93.6%)	25 (78.1%)	0.042
Pulmonary **	13 (27.7%)	19 (59.4%)	0.004
Diabetes mellitus	8 (17.0%)	2 (6.3%)	0.157
Cerebrovascular	3 (6.4%)	2 (6.3%)	0.980
Renal disease	2 (4.3%)	2 (6.3%)	0.691

*, data presented as *n* (%), unless described differently; **, besides COPD; SD, standard deviation; BMI, body mass index; GOLD—The Global Initiative for COPD; COPD, chronic obstructive lung disease; Bonferroni-adjusted *p*-value threshold = 0.0029.

**Table 2:** Patients’ living conditions stratified by COPD severity.

Variables	GOLD 3 (*n* = 47)	GOLD 4 (*n* = 32)	*p*-Value
Place of residence (urban)	39 (83.0%)	22 (68.8%)	0.138
Living area size (mean ± SD)	79.1 ± 42.4	61.3 ± 30.4	0.044
**Living area**			0.017
<30 m^2^	3 (6.4%)	7 (21.9%)	
30–60 m^2^	17 (36.2%)	16 (50.0%)	
>60 m^2^	27 (57.4%)	9 (28.1%)	
**Distance to main road**			0.215
<50 m	19 (40.4%)	7 (21.9%)	
50–200 m	10 (21.3%)	10 (31.3%)	
>200 m	18 (38.3%)	15 (46.9%)	
Distance to main road, meters (mean ± SD)	3823.4 ± 1517.2	800.2 ± 209.5	<0.001
Building height (mean ± SD)	10.6 ± 4.9	10.1 ± 2.6	0.598
**Cooking source**			
Gas	45 (95.7%)	30 (85.7%)	0.107
Electric	2 (4.3%)	1 (2.9%)	0.738
Biomass	2 (4.3%)	4 (11.4%)	0.217
**Type of heating**			
Gas	39 (83.0%)	20 (42.6%)	0.009
Electric	2 (4.3%)	4 (11.4%)	0.217
Biomass	8 (17.0%)	11 (31.4%)	0.126

GOLD, The Global Initiative for COPD; COPD, chronic obstructive lung disease; SD—standard deviation; Bonferroni-adjusted *p*-value threshold = 0.0038.

**Table 3:** Home air measurements stratified by COPD severity.

Variables *	GOLD 3 (*n* = 47)	GOLD 4 (*n* = 32)	*p*-Value
**PM 1.0 (µg/m^3^)**			
Minimum values	3.2 (6.0)	1.3 (5.1)	0.146
Maximum values	105.5 (243.5)	156.7 (273.1)	<0.001
Mean values	15.4 (71.4)	20.1 (30.6)	0.006
**PM 2.5 (µg/m^3^)**			
Minimum values	1.9 (5.8)	3.6 (6.9)	0.168
Maximum values	147.9 (402.1)	140.1 (428.8)	0.493
Mean values	14.9 (38.9)	19.6 (77.2)	0.001
**PM 10 (µg/m^3^)**			
Minimum values	1.6 (6.3)	3.7 (7.3)	0.227
Maximum values	123.8 (350.2)	199.4 (568.2)	<0.001
Mean values	17.5 (38.0)	25.5 (63.4)	<0.001
**Temperature (°C)**			
Minimum values	18.8 (8.2)	21.9 (7.6)	0.116
Maximum values	28.3 (4.1)	29.2 (6.3)	0.304
Mean values	24.6 (5.3)	26.9 (7.6)	0.660
**Humidity (%)**			
Minimum values	39.5 (5.8)	38.5 (7.2)	0.538
Maximum values	56.5 (8.9)	53.5 (9.3)	0.092
Mean values	45.4 (5.8)	43.9 (8.7)	0.274
**Pressure (atm)**			
Minimum values	1007.9 (13.1)	1003.7 (11.6)	0.319
Maximum values	1016.4 (10.3)	1014.9 (8.3)	0.563
Mean values	1011.6 (13.8)	1008.9 (9.0)	0.183

*, data presented as median (IQR); IQR, interquartile range; GOLD, The Global Initiative for COPD; COPD, chronic obstructive lung disease; SD, standard deviation; PM, particulate matter; Bonferroni-adjusted *p*-value threshold = 0.0028.

**Table 4:** Lung function studies and patients’ investigations stratified by COPD severity.

Variables *	GOLD 3 (*n* = 47)	GOLD 4 (*n* = 32)	*p*-Value
Frequent exacerbations, *n* (%)	18 (38.3%)	20 (57.1%)	0.090
Oxygen supplementation at home, *n* (%)	27 (57.4%)	22 (62.9%)	0.621
**Spirometry**			
FEV1 (%)	40.0 (8.9)	23.5 (7.2)	<0.001
FEV1 (L)	1.04 (0.40)	0.65 (0.28)	<0.001
FVC (%)	58.6 (10.5)	40.1 (12.5)	<0.001
FVC (L)	1.91 (0.69)	1.38 (0.49)	<0.001
FEV1/FVC (%)	55.1 (10.8)	44.9 (11.1)	<0.001
FEF 25–75 (L)	0.50 (0.26)	0.31 (0.16)	<0.001
FEF 25–75 (%)	18.3 (10.0)	10.2 (4.3)	<0.001
**COPD assessment**			
CAT	22.9 ± 6.5	25.5 ± 7.4	0.103
mMRC	2.9 ± 0.7	3.2 ± 0.7	0.065
**Other studies**			
Systolic BP (mean ± SD)	130.2 ± 18.9	132.9 ± 16.2	0.512
Diastolic BP (mean ± SD)	79.9 ± 10.4	78.6 ± 10.1	0.582
Creatinine (mean ± SD)	0.8 ± 0.2	0.9 ± 0.3	0.079
BUN (mean ± SD)	41.8 ± 19.2	32.9 ± 12.9	0.024
Glucose (mean ± SD)	114.8 ± 40.4	105.5 ± 35.8	0.297

*, data presented as median (IQR); IQR, interquartile range; GOLD, The Global Initiative for COPD; COPD, chronic obstructive lung disease; SD, standard deviation; CAT, COPD assessment test; mMRC, modified Medical Research Council; FEV1, forced expiratory volume in the first second; FVC, forced vital capacity; FEF25–75%, forced expiratory flow at 25–75% of the pulmonary volume; BP, blood pressure; BUN, blood urea nitrogen; Bonferroni-adjusted *p*-value threshold = 0.0031.

**Table 5:** Risk-factor analysis for worsening COPD.

Variables	β	SE	OR	95% CI	*p*-Value
**GOLD (dependent)**					
PM 1.0 (µg/m^3^)	0.025	0.005	1.39	1.05–2.44	0.002
PM 2.5 (µg/m^3^)	0.009	0.005	1.07	0.96–1.88	0.195
PM 10.0 (µg/m^3^)	0.016	0.007	1.12	1.01–1.63	0.040
Living area size	0.010	0.006	0.46	0.29–0.94	0.010
Distance to main road	0.033	0.004	1.58	1.24–2.16	<0.001
**CAT (dependent)**					
PM 1.0 (µg/m^3^)	0.012	0.007	1.06	1.01–1.43	0.038
PM 2.5 (µg/m^3^)	0.004	0.004	1.01	0.87–1.29	0.206
PM 10.0 (µg/m^3^)	0.008	0.004	1.13	1.06–1.76	0.009
Living area size	0.013	0.008	0.51	0.26–0.92	<0.001
Distance to main road	0.021	0.007	1.30	1.10–1.85	<0.001
**mMRC (dependent)**					
PM 1.0 (µg/m^3^)	0.016	0.006	1.28	1.08–1.92	0.001
PM 2.5 (µg/m^3^)	0.010	0.004	1.10	1.03–1.48	0.018
PM 10.0 (µg/m^3^)	0.008	0.005	1.16	1.06–1.59	0.002
Living area size	0.015	0.007	0.38	0.28–0.71	<0.001
Distance to main road	0.019	0.009	1.23	0.99–1.63	0.092

SE, standard error; OR, odds ratio; CI, confidence interval; PM, particulate matter; CAT, COPD assessment test; mMRC, modified Medical Research Council.

## Data Availability

Data available on request.

## References

[1] Devine J.F. (2008). Chronic obstructive pulmonary disease: An overview. Am. Health Drug Benefits.

[2] Barata P.I., Crisan A.F., Maritescu A., Negrean R.A., Rosca O., Bratosin F., Citu C., Oancea C. (2022). Evaluating Virtual and Inpatient Pulmonary Rehabilitation Programs for Patients with COPD. J. Pers. Med..

[3] Alobaidi N.Y., Stockley J.A., Stockley R.A., Sapey E. (2020). An overview of exacerbations of chronic obstructive pulmonary disease: Can tests of small airways’ function guide diagnosis and management?. Ann. Thorac. Med..

[4] Mathioudakis A.G., Janssens W., Sivapalan P., Singanayagam A., Dransfield M.T., Jensen J.S., Vestbo J. (2020). Acute exacerbations of chronic obstructive pulmonary disease: In search of diagnostic biomarkers and treatable traits. Thorax.

[5] Pescaru C.C., Crisan A.F., Marc M., Trusculescu A.A., Maritescu A., Pescaru A., Sumenkova A., Bratosin F., Oancea C., Vastag E. (2023). A Systematic Review of Telemedicine-Driven Pulmonary Rehabilitation after the Acute Phase of COVID-19. J. Clin. Med..

[6] Xing Y.F., Xu Y.H., Shi M.H., Lian Y.X. (2016). The impact of PM2.5 on the human respiratory system. J. Thorac. Dis..

[7] Were F.H., Wafula G.A., Lukorito C.B., Kamanu T.K.K. (2020). Levels of PM10 and PM2.5 and Respiratory Health Impacts on School-Going Children in Kenya. J. Health Pollut..

[8] Patel A.R., Patel A.R., Singh S., Singh S., Khawaja I. (2019). Global Initiative for Chronic Obstructive Lung Disease: The Changes Made. Cureus.

[9] Yawn B.P., Mintz M.L., Doherty D.E. (2021). GOLD in Practice: Chronic Obstructive Pulmonary Disease Treatment and Management in the Primary Care Setting. Int. J. Chron. Obstr. Pulm. Dis..

[10] Karloh M., Fleig Mayer A., Maurici R., Pizzichini M.M.M., Jones P.W., Pizzichini E. (2016). The COPD Assessment Test: What Do We Know So Far? A Systematic Review and Meta-Analysis About Clinical Outcomes Prediction and Classification of Patients into GOLD Stages. Chest.

[11] Cheng S.L., Lin C.H., Wang C.C., Chan M.C., Hsu J.Y., Hang L.W., Perng D.W., Yu C.J., Wang H.C. (2019). Taiwan Clinical Trial Consortium for Respiratory Disease (TCORE). Comparison between COPD Assessment Test (CAT) and modified Medical Research Council (mMRC) dyspnea scores for evaluation of clinical symptoms, comorbidities and medical resources utilization in COPD patients. J. Formos. Med. Assoc..

[12] Voica A.S., Oancea C., Tudorache E., Crisan A.F., Fira-Mladinescu O., Tudorache V., Timar B. (2016). Chronic obstructive pulmonary disease phenotypes and balance impairment. Int. J. Chron. Obstr. Pulm. Dis..

[13] Kim S., Oh J., Kim Y.I., Ban H.J., Kwon Y.S., Oh I.J., Kim K.S., Kim Y.C., Lim S.C. (2013). Differences in classification of COPD group using COPD assessment test (CAT) or modified Medical Research Council (mMRC) dyspnea scores: A cross-sectional analyses. BMC Pulm. Med..

[14] Crişan A.F., Oancea C., Timar B., Fira-Mladinescu O., Tudorache V. (2015). Balance impairment in patients with COPD. PLoS ONE.

[15] Natori H., Kawayama T., Suetomo M., Kinoshita T., Matsuoka M., Matsunaga K., Okamoto M., Hoshino T. (2016). Evaluation of the Modified Medical Research Council Dyspnea Scale for Predicting Hospitalization and Exacerbation in Japanese Patients with Chronic Obstructive Pulmonary Disease. Intern. Med..

[16] Schraufnagel D.E. (2020). The health effects of ultrafine particles. Exp. Mol. Med..

[17] Dondi A., Carbone C., Manieri E., Zama D., Del Bono C., Betti L., Biagi C., Lanari M. (2023). Outdoor Air Pollution and Childhood Respiratory Disease: The Role of Oxidative Stress. Int. J. Mol. Sci..

[18] Mukherjee S., Dasgupta S., Mishra P.K., Chaudhury K. (2021). Air pollution-induced epigenetic changes: Disease development and a possible link with hypersensitivity pneumonitis. Environ. Sci. Pollut. Res. Int..

[19] Mack S.M., Madl A.K., Pinkerton K.E. (2019). Respiratory Health Effects of Exposure to Ambient Particulate Matter and Bioaerosols. Compr. Physiol..

[20] Crişan A.F., Oancea C., Timar B., Fira-Mladinescu O., Crişan A., Tudorache V. (2014). Cognitive impairment in chronic obstructive pulmonary disease. PLoS ONE.

[21] Burkes R.M., Gassett A.J., Ceppe A.S., Anderson W., O’Neal W.K., Woodruff P.G., Krishnan J.A., Barr R.G., Han M.K., Martinez F.J. (2018). Current and former investigators of the SPIROMICS sites and reading centers. Rural Residence and Chronic Obstructive Pulmonary Disease Exacerbations. Analysis of the SPIROMICS Cohort. Ann. Am. Thorac. Soc..

[22] Barata P.I., Marc M.S., Tudorache E., Frandes M., Crisan A.F., Olar D.C., Oancea C. (2021). Self-reported sleep disturbance and mild cognitive impairment in COPD patients with severe airflow limitation. Clin. Respir. J..

[23] Vimercati L. (2011). Traffic related air pollution and respiratory morbidity. Lung India.

[24] Hogea P., Tudorache E., Fira-Mladinescu O., Marc M., Velescu D., Manolescu D., Bratosin F., Rosca O., Mavrea A., Oancea C. (2023). Serum and Bronchoalveolar Lavage Fluid Levels of Cytokines in Patients with Lung Cancer and Chronic Lung Disease: A Prospective Comparative Study. J. Pers. Med..

[25] Pacurari A.C., Bhattarai S., Muhammad A., Avram C., Mederle A.O., Rosca O., Bratosin F., Bogdan I., Fericean R.M., Biris M. (2023). Diagnostic Accuracy of Machine Learning AI Architectures in Detection and Classification of Lung Cancer: A Systematic Review. Diagnostics.

[26] Jiao Y., Gong C., Wang S., Duan Y., Zhang Y. (2022). The Influence of Air Pollution on Pulmonary Disease Incidence Analyzed Based on Grey Correlation Analysis. Contrast Media Mol. Imaging.

[27] Jiang X.Q., Mei X.D., Feng D. (2016). Air pollution and chronic airway diseases: What should people know and do?. J. Thorac. Dis..

[28] Nazar W., Niedoszytko M. (2022). Air Pollution in Poland: A 2022 Narrative Review with Focus on Respiratory Diseases. Int. J. Environ. Res. Public Health.

[29] Güder G., Brenner S., Angermann C.E., Ertl G., Held M., Sachs A.P., Lammers J.W., Zanen P., Hoes A.W., Störk S. (2012). GOLD or lower limit of normal definition? A comparison with expert-based diagnosis of chronic obstructive pulmonary disease in a prospective cohort-study. Respir. Res..

[30] Velea L., Udriștioiu M.T., Puiu S., Motișan R., Amarie D. (2023). A Community-Based Sensor Network for Monitoring the Air Quality in Urban Romania. Atmosphere.

[31] Gil H.I., Zo S., Jones P.W., Kim B.G., Kang N., Choi Y., Cho H.K., Kang D., Cho J., Park H.Y. (2021). Clinical Characteristics of COPD Patients According to COPD Assessment Test (CAT) Score Level: Cross-Sectional Study. Int. J. Chron. Obstr. Pulm. Dis..

[32] Pisi R., Aiello M., Calzetta L., Frizzelli A., Tzani P., Bertorelli G., Chetta A. (2023). The COPD assessment test and the modified Medical Research Council scale are not equivalent when related to the maximal exercise capacity in COPD patients. Pulmonology.

[33] Atkinson R.W., Carey I.M., Kent A.J., Van Staa T.P., Anderson H.R., Cook D.G. (2015). Long-term exposure to outdoor air pollution and the incidence of chronic obstructive pulmonary disease in a national English cohort. Occup. Environ. Med..

[34] Schikowski T., Sugiri D., Ranft U., Gehring U., Heinrich J., Wichmann H., Krämer U. (2005). Long-term air pollution exposure and living close to busy roads are associated with COPD in women. Respir. Res..

[35] Liu S., Zhou Y., Liu S., Chen X., Zou W., Zhao D., Li X., Pu J., Huang L., Chen J. (2017). Association between exposure to ambient particulate matter and chronic obstructive pulmonary disease: Results from a cross-sectional study in China. Thorax.

[36] Ko F.W., Hui D.S. (2012). Air pollution and chronic obstructive pulmonary disease. Respirology.

[37] Rice M.B., Ljungman P.L., Wilker E.H., Dorans K.S., Gold D.R., Schwartz J., Koutrakis P., Washko G.R., O’Connor G.T., Mittleman M.A. (2015). Long-term exposure to traffic emissions and fine particulate matter and lung function decline in the Framingham Heart Study. Am. J. Respir. Crit. Care Med..

[38] Rice M.B., Rifas-Shiman S.M., Litonjua A.A., Oken E., Gillman M.W., Kloog I., Luttmann-Gibson H., Zanobetti A., Coull B.A., Schwartz J. (2015). Lifetime exposure to ambient pollution and lung function in children. Am. J. Respir. Crit. Care Med..

[39] Kariisa M., Foraker R., Pennell M., Buckley T., Diaz P., Criner G.J., Wilkins J.R. (2015). Short- and long-term effects of ambient ozone and fine particulate matter on the respiratory health of chronic obstructive pulmonary disease subjects. Arch. Environ. Occup. Health.

[40] Lepeule J., Litonjua A.A., Coull B., Koutrakis P., Sparrow D., Vokonas P.S., Schwartz J. (2014). Long-term effects of traffic particles on lung function decline in the elderly. Am. J. Respir. Crit. Care Med..

[41] Schikowski T., Adam M., Marcon A., Cai Y., Vierkötter A., Carsin A.E., Jacquemin B., Al Kanani Z., Beelen R., Birk M. (2014). Association of ambient air pollution with the prevalence and incidence of COPD. Eur. Respir. J..

[42] Saha L., Kumar A., Kumar S., Korstad J., Srivastava S., Bauddh K. (2022). The impact of the COVID-19 lockdown on global air quality: A review. Environ. Sustain..

